# Intensive use of IVF by large-scale dairy programs

**DOI:** 10.21451/1984-3143-AR2019-0058

**Published:** 2019-10-23

**Authors:** Bruno Valente Sanches, Amanda Fonseca Zangirolamo, Marcelo Marcondes Seneda

**Affiliations:** 1 Vytelle IVF, LLC, Hermiston, OR, USA.; 2 Universidade Estadual de Londrina, Laboratório de Reprodução Animal, DCV-CCA-UEL, Londrina, Parana, Brazil.; 3 National Institute of Science and Technology for Dairy Production Chain (INCT–LEITE), Universidade Estadual de Londrina, Rodovia Celso Garcia Cid-Campus Universitário, Parana, Brazil.

**Keywords:** IVF, bovine, dairy, commercial use, genomic analysis

## Abstract

The number of embryos produced by *in vitro* fertilization (IVF) has grown exponentially in recent years. Recently, for the first time, the number of embryos produced and transferred *in vitro* was significantly higher than the number developed *in vivo* worldwide. In this context, a particular boost occurred with ovum pick-up (OPU) and *in vitro* embryos produced in North America, and this technology is becoming more prominent for commercial dairy farms. However, despite many advances in recent decades, laboratories and companies are looking for methods and alternatives that can be used in collaboration with the existing process to improve it. Among the strategies used to improve the dairy industry are the use of genomic analysis for the selection of animals with desired traits or as an evaluation tool of oocyte and embryo quality, the optimization of the collection and use of gametes from prepubertal females and males, the effective use of sexed semen, and improvements in culture media and methods of embryo cryopreservation. Thus, this review aims to discuss the highlights of the commercial use of IVF and some strategies to increase the application of this technique in large-scale dairy programs.

## Introduction

The dairy industry plays an essential role in the global socioeconomic scenario. Although growth in global milk production has been limited in recent years, it is projected to increase by 22% in 2027 compared to 2015-2017 (OECD and FAO, 2018). The dairy industry is the leader among the food animal sector in the successful application of advanced technologies (Wiggans *et al*., 2017). Therefore, practices and alternatives that improve the production of dairy cattle are increasingly required.

The increase in the productive efficiency and quality of animal products from livestock has been possible due to the use of reproductive biotechniques (Hansen *et al*., 2014). In this context, *in vitro* fertilization (IVF) is a useful tool when performing the selection and breeding of genetically superior animals (Hansen *et al*., 2014), which is becoming more prominent in commercial dairies (Sirard, 2018).

**Figure 1:**
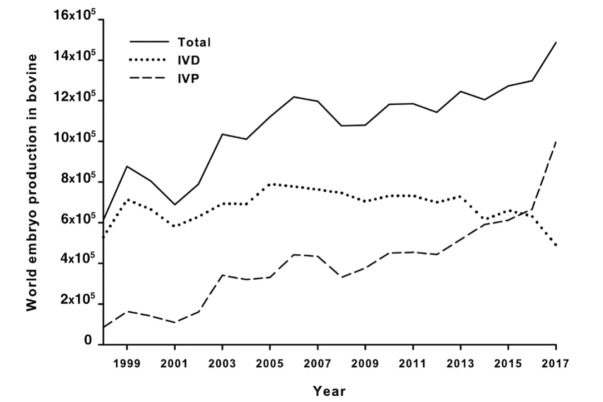
The number of bovine embryos produced (*in vivo* - IVD, *in vitro* - IVP, and total) recorded in the period 1998 - 2017 (Data sourced from Viana, 2017; Viana, 2018).

**Figure 2:**
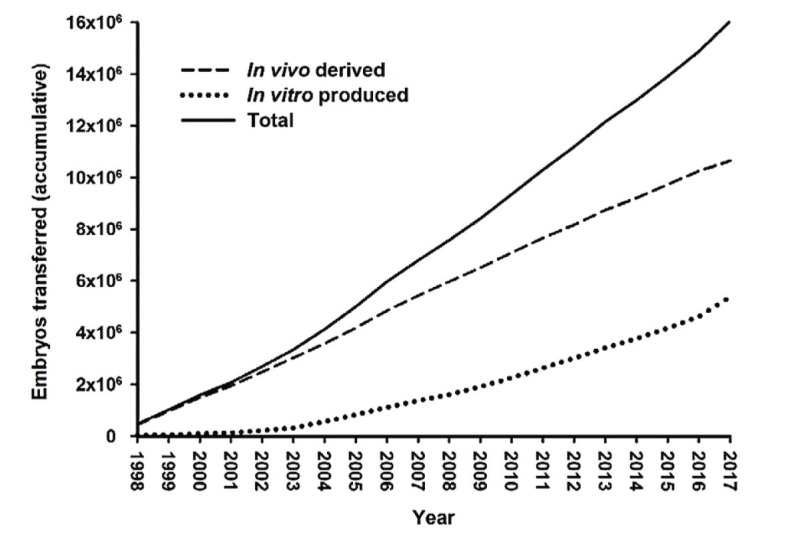
The accumulated number of bovine embryos transferred in the period 1998 - 2017, based on *in vivo* or *in vitro* production methods (Data sourced from Viana, 2017; Viana, 2018).

According to the Embryo Transfer Newsletter (Viana, 2017), for the first time, the number of bovine embryos produced and transferred *in vitro* was significantly higher than those *in vivo* produced worldwide (Figs. 1-2; Viana, 2017).

**Figure 3:**
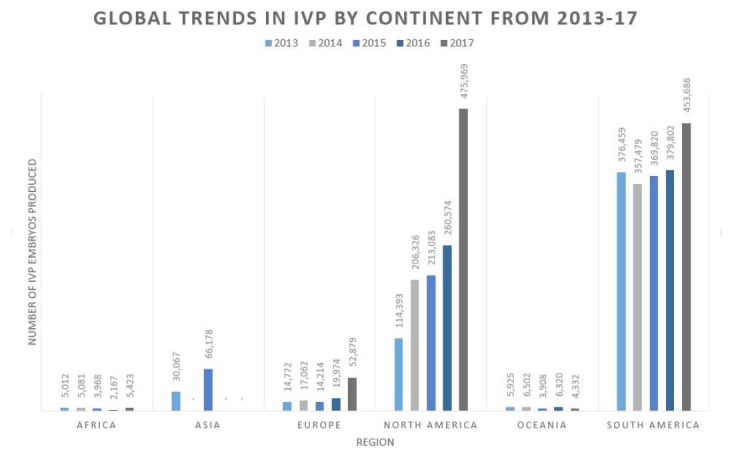
The accumulated number of IVP bovine embryos in the period 2013 - 2017 by continent (Data sourced from Viana, 2017; Viana, 2018).

Furthermore, for the first time since 1999, North America has reported more *in vitro* produced (IVP) embryos than South America, the region that led the use of IVF in the past decade (Fig. 3; Viana, 2018). Notably, the United States (US) had the highest number of IVP embryos within North America, at approximately 95.5% (Viana, 2018). The further development of the embryo industry in North America seems to resemble what happened in South America, in which the contribution of *in vivo* embryos has been linear, and the use of IVP embryos has resulted in a substantial increase in numbers (Viana *et al*., 2018).

However, despite advances in IVF, the embryo production rate from the total cumulus oocyte complexes (COC), the embryo production rate remains at 30 to 40% (Lonergan *et al*., 2016). Thus, laboratories and companies have been looking for alternatives that collaborate to improve the existing process and to optimize methods to use IVF in large-scale dairy programs.

The genomic testing of cattle is now significantly affecting IVF programs. Genomic selection has revolutionized dairy farming, shortening the breeding interval, increasing selection accuracy, and reducing the previous costs of progeny testing (Wiggans *et al*., 2017). The commercial interest in performing genomic analysis and collecting gametes from prepubertal animals that have desired traits is increasing (Moore and Hasler, 2017). The small ultrasound OPU probes currently available allow IVP embryos from younger females to be grown (Sirard, 2018). Additionally, genomic analysis has been used to evaluate the quality and viability of oocytes, and even the embryos, before transfer procedures (Moore and Hasler, 2017).

An embryo culture media have been developed to mimic what happens in the maternal organism. Several studies have been performed to investigate the addition of different products and molecules in the culture medium, such as cytokines, growth factors, and antioxidants, and many advances have been obtained.

Sexing technology is another practice used to improve IVF results.; The use of sexed semen enables the birth of offspring of a predetermined sex, as well as increase the efficiency of producing donors with the right genetic background. Furthermore, IVF is the mostcommon application of sexed semen, which has superior efficacy compared with its use in other areas of biotechnology (Morotti *et al*., 2014).

Due to the increasing number of IVP embryos, cryopreservation methods provide a good alternative for the storage of surplus products. However, some limitations exist, which may hamper the use of cryopreservation on a large scale. In this context, among the different protocols, the process of thawing and the direct transfer of embryos together make the cryopreservation protocol more efficient for commercial use by facilitating logistics in the field (Sanches *et al*., 2016).

Thus, this review aims to discuss some strategies to increase the useful application of IVF in large-scale dairy programs, as well as the trends, challenges, and highlights of the commercial use of the IVF program.

## Genomic analysis: from animal selection to oocyte and embryo evaluation

Currently, genomic analysis is driving the development of several IVF laboratories in North America and other places in the world (Sirard, 2018). The best effect of genomic selection to date has been to double the rate of genetic progress for traits of economic interest. Genetic improvement occurs through the increased accuracy of genetic merit for young animals (Wiggans *et al*., 2017).

With genomic analysis performed soon after birth, the genetic value of the bull is determined early, and as soon as semen is produced, such high genetic merit sperm, can be used for IVF. Moreover, it has increased the commercial demand for producing embryos from young heifers and calves (Sirard, 2018). Currently, the collection of oocytes from donors before puberty is possible with relatively high success (Landry *et al*., 2016). Additionally, genomic selection is helpful for choosing better embryo recipients according to the genes involved with gestation maintenance.

**Figure 4:**
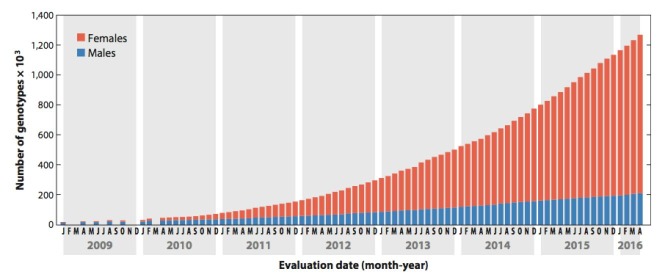
The number of genotyped animals included in US genomic evaluations for dairy cattle since January 2009 (Data sourced from the Council on Dairy Cattle Breeding, 2016; Wiggans *et al*., 2017).

Genomic evaluations for Holsteins, Jerseys, and Brown Swiss became official in 2009 at the USDA, and since that time, more than 1 million animal genotypes have received genetic evaluations (Council on Dairy Cattle Breeding, 2016). Due to the popularity of genotyping chips, microsatellites have been replaced by SNPs, and the accessibility to chips of lower cost has made whole herd genotyping common in the US (Wiggans *et al*., 2017). The scenario of genotyped animals included in US genomic evaluations for dairy cattle is shown in Fig. 4.

**Figure 5:**
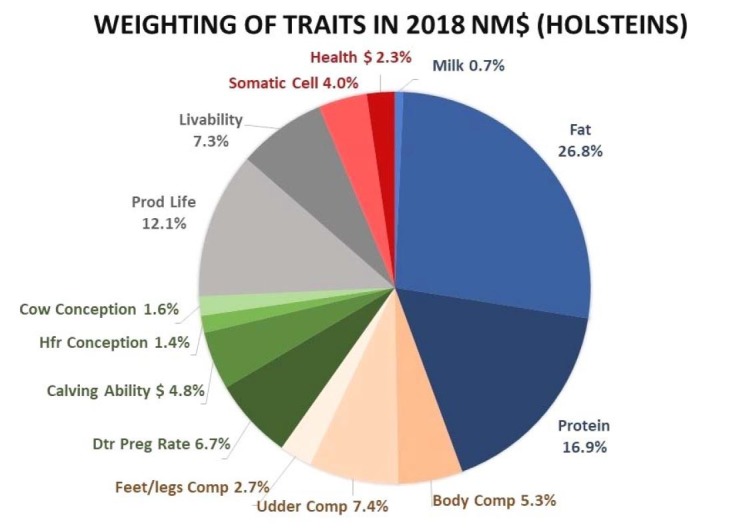
Composition and weighting of the 14 traits in 2018 Net Merit. (Available on: https://hoards.com/article-23717-net-merit-$-index-updated-to-include-health-traits.html. Accessed on April 10, 2019).

Additionally, dairy cattle can be selected for any combination of traits, but total genetic progress will be fastest using an index because many traits affect profitability. In this context, the lifetime net merit (NM$) index, elaborated by the U.S. Department of Agriculture (USDA), ranks dairy animals based on their combined genetic merit for economically important traits (VanRaden, 2018). The NM$ index includes economically important traits related to health, yield, longevity and calving ease, and because it is calculated using Holstein values, it is, therefore, widely used for this breed (VanRaden, 2018). The weighting and composition of the 14 traits that make up the net merit in the year 2018 are shown in Fig. 5.

However, the relative importance of traits differed slightly between the production systems. Organic dairy producers, for example, tend to prefer health traits as the pillar of selection, even though the increase in the genetic gain in disease resistance is achieved at the expense of milk production, since they cannot use any medicine or chemical in animals (Fall *et al*., 2008). Moreover, the CM$ provides longevity and somatic cell score data for producers whose milk is made into cheese or other dairy products (VanRaden, 2017). Although the Jersey produces less milk than the Holstein, it produces milk with more fat, milk protein, and a higher energy content (Aikman, *et al*., 2007). Therefore, for Jersey cows, the CM$ would possibly be more interesting.

Another application of genomic analysis is the use of micro-array or RNAseq technologies on embryos submitted to different culture conditions, with the intention of comparing *in vivo* control embryos and, thus, improving culture methods (Sirard, 2018). Furthermore, embryo culture medium can provide a source of material for noninvasive embryonic genetic testing (without biopsies, for example). However, whether the DNA found represents the genetic state of the embryo remains unknown. Thus, this potentially noninvasive approach must be validated by additional experiments (Liu *et al*., 2017; Smith *et al*., 2019), and once confirmed, it can lead to other methods to evaluate the quality of embryos.

## Strategies to improve oocyte competence before OPU

The quality of the oocyte is the central factor interfering with the blastocyst yield, as well as the potential explanation for the limited success rates of IVF (Lonergan and Fair, 2016). The oocyte competence, and consequently the development to the blastocyst stage, is positively associated with the size of the antral follicle (Lonergan *et al*., 1994) and whether the blastocyst was produced *in vivo* or *in vitro* (Rizos *et al*., 2002).

One strategy for manipulating follicular growth and affecting developmental competence is Coasting, which is a period between hormonal stimulation and ovary collection (Nivet *et al*., 2012). In adult females, this approach allowed a high rate of blastocyst development after IVF, suggesting an increase in oocyte quality (Blondin *et al*., 2002). Animals that received sixinjections of FSH, followed by a 48-h coasting period and an injection of LH 6 h before ovum pick-up (OPU), presented an 80% rate of blastocyst stage occurrence (Blondin *et al*., 2002). More recently, the same group showed that the ideal period was 54 ± 7 hours, where a well-defined period of competence to recover oocytes of the highest quality is of paramount importance (Nivet *et al*., 2012).

During OPU oocytes are recovered on random days of the estrous cycle, i.e., follicles that are at different stages of development (Wit *et al*., 2000). In these conditions, more than 85% of the aspirated ovarian follicles present some degree of atresia due to the apoptosis process (Hendriksen *et al*., 2000). Recently, follicular wave synchronization before OPU was observed to provide an increase in embryo production rates and post-transfer conception for the recipients (Cavalieri *et al*., 2018).

## Collection and quality of oocytes from prepubertal heifers and calves

Recently, there has been an increase in the commercial interest in producing bovine embryos from prepubertal heifers and calves. The interest in breeding the best animals at younger ages is to accelerate the genetic advancement rate of genetic gain (Baldassarre *et al*., 2018). With the emergence of genomic technologies in recent years, the prediction of better phenotype production has been possible after birth of the animal (Ponsart *et al*., 2013).

In the early 1990s, the development of transvaginal oocyte recovery procedures in cattle improve the IVF method (Pieterse *et al*., 1991). Initially, due to animal size issues, the collection of oocytes by OPU was very difficult or not possible, and laparoscopic ovum pick-up (LOPU) rapidly became the method of choice for small animals such as calves and pre-pubertal heifers (Cognie *et al*., 2004). Currently, small ultrasound OPU probes are available and allow IVP embryos from younger females to be grown (Moore and Hasler, 2017).

Several studies have shown that bovine calf oocytes are significantly less capable of developing into embryos compared with oocytes from adult cows (Baldassarre and Bordignon, 2018). Prepubertal females have immature and nonfunctional hypothalamus-pituitary-ovarian axes and, therefore, are unable to achieve full follicular development and ovulation (Sanchez and Smitz, 2012). Thus, different research groups are directing efforts seeking to improve quality and increase oocyte competence in young heifers.

Studies described the recovery of a high number of oocytes from females 2-6 months of age who were stimulated with gonadotropins to increase the proportion (and size) of large follicles (Baldassarre and Bordignon, 2018). In some cases, the number of oocytes was higher than what was recovered from adult cows.

*B. taurus* and *B. indicus* aging from 2 to 4 months did not exhibit an improvement in IVF results when stimulated with 140 mg of FSH (Batista *et al*., 2016). However, recently, more prolonged FSH stimulation (three days) was shown to increase the development competence of Holstein calf oocytes, which was associated with a higher proportion of follicles larger than 5 mm (Currin *et al*., 2017).

A previous study showed that Holstein calves aging from 5 to 7 months had more oocytes than cycling heifers aging from 16 to 18 months. Although the blastocyst rate was higher in the cycling heifers than in the calves, the number of embryos (6–8) was not different (Landry *et al*., 2016). Further studies are necessary to investigate the beneficial effects of exogenous gonadotropins to prepubertal heifers and calves.

Other possible future efforts of research include the development of *in vitro* maturation (IVM) protocols with strategies for delaying nuclear and improving cytoplasmic maturation. Additionally, another research target includes the supplementation of IVM medium with substances or molecules that improve oocyte development for step embryo transfer (Baldassarre and Bordignon, 2018). We also believe that the epigenetic changes or even the nutrition of the mother, which can interfere in the quality of the oocytes of the daughters, will be an area for future research.

Thus, with the advent of genomic analysis, the extraordinary growth of IVF technologies in recent years and high interest by dairy producers and the use of elite females that are as young as possible (from 2 months of age) for embryo production has the potential to help IVF become a viable practice very soon.

## Advances in embryo culture media

For *in vitro* embryo production (IVEP), specific media are used for maturation, fertilization, and *in vitro* culture to mimic what occurs physiologically in the organism. In cattle, approximately 90% of immature oocytes, recovered from follicles at unknown stages of the estrous cycle (ovaries from slaughterhouse), undergo nuclear maturation *in vitro* and approximately 80% undergo fertilization (Lonergan and Fair, 2016).

The media used may be a determinant factor in the production and quality of blastocysts and embryo cryotolerance (Sanches *et al*., 2013). Changes in the culture conditions such as the addition of lipolytic chemical substances and the adjustment of fetal calf serum in the medium have been proposed to increase the embryo cryotolerance (Sanches *et al*., 2017). In this context, several studies show forskolin and phenazine ethosulfate (PES), as substances which reduces lipid accumulation (Sudano *et al*., 2011; Paschoal *et al*., 2017).

Although modest improvements have occurred in the development and composition of IVM media (addition of different products, cytokines, growth factors, antioxidants, and other substances), the blastocyst rate rarely exceeds 40–50% (Lonergan and Fair, 2016). Thus, the yield of oocytes developing to the blastocyst stage remains very similar to that in the years 1990 to 2000, in which it reached a plateau at 30-40% (Sirard, 2018).

Another strategy to improve embryo culture media is to try to keep what occurs physiologically in the follicular environment of the oocyte, in which the arrest of meiosis is maintained. Several meiotic inhibitors can delay the resumption of *in vitro* meiosis. Thereby, the continuous accumulation of mRNA and proteins within the oocyte allows a better cytoplasmic maturation (Bilodeau-Goessels, 2012).

However, despite the many protocols and tested methods *in vitro*, attention has turned toward the source of oocytes as the cause of the limited success rates of IVF (Sirard, 2018).

## Use of sexed semen and its advantages

In the dairy industry, the production of overweight calves from undesirable sex (i.e. male) is a particularly important issue (Holden and Butler, 2018). The use of sexed semen in association with reproductive biotechnologies represents a significant advance in the global livestock industry. In this context, the predetermination of the sex of the animal optimizes production and profitability in dairy herds (Morotti *et al*., 2014).

Among the reproductive biotechnologies, the most common application of sexed semen is IVF due to good blastocyst rates can be achieved (Matoba *et al*., 2014). Moreover, IVF to require far less sperm per oocyte to make acceptable fertilization rates compared with AI (Holden and Butler, 2018). In the US, more than 90% of 4.5 to 5 million straws of sexed semen were from milk dairy bull sires in 2016 (Moore and Hasler, 2017).

However, studies show that the blastocyst rates are lower than those obtained with conventional semen (Seidel Jr. 2014). On the other hand, Cottle *et al*. (2018) identified a significant profit advantage for using sexed semen in the context of a high-output, dairy system of spring births in Ireland. The authors concluded that the use of sexed semen is more appropriate for those farms that already have an excellent fertility performance. Thus, the lowest rates associated with sexed semen can be less acceptable for farms with sub-optimal dairy herds fertility (Cottle *et al*., 2018).

In any case, genetic targeting of the dairy herd to achieve desired sex animals justifies the expansion of the use of sexed semen in the dairy sector.

## Advances in cryopreservation with the use of DT

Despite the IVF advantages, cryopreservation represents a challenge for commercial laboratories. The low cryotolerance of IVP embryos is a limiting factor to the use of the cryopreservation process in an IVF program (Sudano *et al*., 2011). In addition, after the cryopreservation is well established, we believe the number of field technicians trained to do the transfer process will not be sufficient. Thus, efforts should be made to overcome all limitations involving the use of IVP embryo cryopreservation on a large scale and globally.

Among cryopreservation techniques, vitrification is more often used worldwide due to the speed and low cost (Dode, 2013). However, its method requires a high concentration of cryoprotectants and a trained professional to evaluate embryo quality prior to the transfer (Vajta and Kuwayama, 2006).

In contrast, the direct transfer (DT), a method used since the 1990s to simplify the post-thawing rehydration step of *in vivo* embryos, has been proving to be a useful alternative for commercial use in IVP embryos.

In a study with Girolando donors (1/2 Gir and 1/2 Holstein), the conception rates obtained were 51.35 ± 1.87% (133/259) for the fresh embryos, 35.89 ± 3.87% (84/234) for the vitrified embryos, and 40.19 ± 4.65% (125/311) for the embryos submitted to DT (Sanches *et al*., 2016). Possibly, IVP embryos with sexed semen could be directly transferred with similar conception rates to those submitted to vitrification.

The low concentration of cryoprotectants is the main advantage of this technique because of the reduced toxicity to the embryos (Voelkel and Hu, 1992). Furthermore, the DT eliminates the evaluation before transfer and, therefore, is more practical than vitrification (Sanches *et al*., 2017). Finally, due to the promising results, DT has been implemented in large-scale operations, mainly in the US and Brazil.

## Final comments

The genomic selection of young animals, associated with sexed semen and frozen IVP-blastocysts and following direct transfer protocols, is driving a new era of IVF in the dairy sector (Sirard, 2018). However, since many of these processes are sensitive to operators or even the environment, the challenge of making IVF fully business-grade remains.

In the US, some large dairies have already left behind the commercially available genomic tests and have begun to implement their own genomic assessments and methods for identifying the best animals to be reproduced. Therefore, this behavior of the industry indicates that companies in the business of IVF also need to invest in innovation to develop a more personalized product because, ultimately, they must go beyond the goal of delivering a quality embryo and/or ensuring a high pregnancy rate.
